# Cost of care and social consequences of very low birth weight infants without premature- related morbidities in Italy

**DOI:** 10.1186/s13052-015-0165-z

**Published:** 2015-08-19

**Authors:** Maria Caterina Cavallo, Attilio Gugiatti, Giovanni Fattore, Simone Gerzeli, Dario Barbieri, Rinaldo Zanini, on behalf of the Neonatal Adequate Care for Quality of Life (NEO-ACQUA) Study Group

**Affiliations:** Centre for Research on Health and Social Care Management (CERGAS), Bocconi University, Via Roentgen 1, 20136 Milan, Italy; Department of Political and Social Sciences, University of Pavia, Corso Strada Nuova 65, Pavia, Italy; NICU, Manzoni Hospital, Via dell’Eremo 9/11, Lecco, Italy

## Abstract

Aim of this study was to estimate the cost that is borne by the Italian National Health Service, families, and social security due to very low birth weight infants (VLBWIs) without prematurity-related morbidities up to the age of 18 months. We followed up on 150 VLBWIs and 145 comparable full-term infants (FTIs) who were born in one of 25 different neonatal intensive care units upon discharge from the hospital and at six and 18 months of age. The average length of the primary hospitalisation of the VLBWIs was 59.7 days (SD 21.6 days), with a total cost of €20,502 (SD €8409), compared with three days (SD 0.4 days) with a total cost of €907 (SD €304) for the FTIs. The total societal cost of the VLBWIs for the first 18 months of life was €58,098 (SD €21,625), while the corresponding figure for FTIs was €24,209 (SD €15,557). Among VLBWIs, both low birth weight and gestational age were correlated with the length of hospitalisation after birth (r^2^ = 0.61 and r^2^ = 0.57, respectively; *p* values < 0.0005). Our findings highlight that the existing DRGs and tariffs inadequately reflect the actual costs for Italian National Health Service.

## Background

Approximately 552,000 infants are born in Italy each year, and 1 % of them are very low birth weight infants (VLBWIs) [1], with a birth weight under 1500 g (401 ≤ birth weight ≤1500) or a gestational age (GA) under 30 weeks (22 ≤ GAweeks ≤30). With increasing earlier use of antenatal corticosteroids [2], assisted ventilation, and surfactant [3] and with changing attitudes [4] towards intensive care, survival rates for very preterm births, especially those infants born before 28 weeks of gestation, improved strikingly by the mid 1990s [5–7]. However, preterm birth is still associated with an increased risk of developing a broad range of short-term and long-term complications compared to full-term birth [8, 9]. A substantial body of literature has reported that very low birth weight infants are at an increased risk of negative outcomes, including motor and sensory impairment [10], learning difficulties [11–14] and behavioural problems [15–18]. The care of VLBWIs demands extensive resources, including considerable hospital costs at birth, as previous studies have demonstrated [19–22], and the use of hospital resources remains higher for VLBWIs in early childhood compared to their full-term peers [19, 23–26]. The economic consequences of very preterm birth to society need to be further investigated. Only a few studies have explored the health care and non-health care costs of very preterm children after the first year of life and compared these costs with the costs associated with children of normal birth weight [19]. Although there is evidence that hospital costs represent a considerable portion of the total cost, families also suffer direct economic losses, such as those losses resulting from paying uncovered drugs, travelling costs, or reduced earnings. These costs are rarely investigated.

The aim of this study was to assess the societal cost of the care for the VLBW population born in Italy up to the age of 18 months after correcting for prematurity and to compare these costs to the costs of healthy normal birth weight infants. To reach this aim we used a sample of 150 VLBWIs without premature-related morbidities.

## Methods

### Study design

This study is part of a multicentre, incidence-based, longitudinal study labelled *Neonatal Adequate Care for Quality of Life* (NEO-ACQUA). This is the first Italian study to longitudinally follow a cohort of 178 VLBWIs matched to 178 healthy, full term infants (FTIs) from birth to evaluate their clinical outcomes, the quality of life for their parents, and the cost involved in their care. The study protocol and the first results were previously published [27].

The VLBWIs were enrolled in neonatal intensive care units (NICUs) of 25 Italian hospitals from January 1, 2006 to December 31, 2006. Only level III NICUs were included in the study [27]. The infants were recruited consecutively, and the inclusion criteria were as follows: gestational age ≤ 30 weeks and/or birth weight ≤ 1500 g; no documented neurological pathology, as shown by negative cerebral ultrasound (periventricular leukomalacia up to stage 1); intraventricular haemorrhage up to stage 1 or 2; no sensory deficit (retinopathy up to stage 1 or 2); neonatal hearing screening through auditory brainstem response or otoemissions within the norm at the 34th week; and no malformation syndromes and/or major malformations.

For each VLBWI, a gender-matched full-term infant with a gestational age between 37 and 42 weeks and a 5-min Apgar score ≥ 8 was enrolled at the same hospital. In addition, the full-term infant’s mother was the same age (+/− 5 years) and the parents belonged to the same Hollingshead social class as those parents of the VLBWI [28]. This social classification uses both parents occupational group for a scale from 0 (occupations that do not require an academic foundation) to 90 (occupations that require highly specialised education and training). The gestational age was determined by an ultrasound examination for VLBWIs and was calculated from the last menstrual period for controls. For both VLBWIs and controls, the maternal inclusion criteria were: Italian nationality, maternal age over 18 years, no manifest psychiatric and cognitive pathology and no evidence of drug addiction. This study was approved by the ethics committees of the participating hospitals, and written consent to participate in the study was obtained from all infants’ parents.

The use of resources was documented through ad hoc questionnaires administered to clinicians and parents. The questionnaires were tested in a pilot study with 10 clinicians and parents. Because this study was conducted from the societal perspective, we collected and classified resource consumption as follows: (i) in-hospital healthcare costs, (ii) other healthcare costs borne by the Italian National Health Service (SSN), (iii) health care and non-healthcare costs directly paid by the families of the infants, and (iv) working days lost by parents due to their infant’s birth.

### Study instruments and identification of costs

The background data from the perinatal period were collected by clinicians using each infant’s medical records. Use of resources was collected through 3 interviews to parents conducted at the end of each relevant period: from birth to discharge from hospital (T_0_), from birth to the corrected age of six month (T_6_), and from corrected age of six months to the corrected age of 18 months (T_18_).

To calculate the cost of each admission to the hospital upon birth, we prospectively collected data on the use of drugs, laboratory and imaging tests; days of parenteral nutrition; and transfusions for each infant. For each VLBWI, we consolidated these costs according to prices (drugs) and national tariffs (tests, parenteral nutrition, and transfusions). To these costs, we added the cost of the hospital stay on the basis of a per diem value. The average cost per NICU day was calculated as the total cost for personnel, medical devices and equipment for the entire NICU divided by the total number of NICU days delivered in 2008. Finally, to include overheads, we added to these figures a fixed percentage of direct cost estimated with the help of the hospital administrative departments. The cost of the initial hospitalisation for the control infants were based on the existing fee reimbursed by the regional authority to hospitals for a healthy newborn infant, which was assumed to be a proxy for the actual cost.

Information from the parents concerning their travel distance to the birth hospital, frequency of visits and working hours lost during the initial hospitalisation was collected for both groups.

At six and 18 months, the following data were collected from the parents: infant’s re-hospitalisations, outpatient visits, diagnostic tests (laboratory and imaging tests), drugs, rehabilitation therapies, psychological support, paid care and informal care. The parents also reported any out-of-pocket payments for each of these items. The monetary value attributed to the outpatient care (visits, tests, rehabilitation, drugs) provided by the SSN was calculated using the standard tariffs used to pay providers.

Travel costs were calculated based on the information collected from the parents about their travelling distances and frequency of visits. The amount spent was calculated on the basis of the available average cost per km by car [29] and by train [30] in Italy.

The parents were also requested to detail working days lost during the period after the infant’s birth and to indicate if they had abandoned their job. Their wages were used to evaluate their loss of production on the assumption that their earnings reflect their productivity. The estimated losses were based on the average daily wages for each parent’s occupational group [31]. In Italy, social security support maternity for a period of five months (two months before birth and three months after). After this period, parental leave is available for either of the parents until the child reaches an age of three years (a maximum of six months leave can be taken during the three years). During childcare leave, the parent who stays home is paid financial support depending on their income level. We separated productive loss figures based on whether the parents were compensated by social insurance.

## Statistical analyses

The costs are presented as the means and standard deviations from the means. Analysis of variance was used to detect cost differences between the groups. *P* < 0.5 was considered significant. 95 % CI was also calculated for socio-demographic variables.

We performed *χ*^2^ tests on dichotomised variables; analyses of variance were used to compare means (costs). All costs are expressed in 2008 Euros and the discount rate was 3 % per year. The analyses were performed with Stata 9 (Stata Corp., College Station, TX).

## Results

For 150 eligible VLBWIs and 145 eligible FTIs, data were collected for the entire study period. The demographic characteristics of both groups are shown in Table 1. They were very similar in terms of gender and the mother’s education in years. They differed slightly in terms of the father’s education years (12.5 years in the VLBWI group vs. 11.5 years in the control group) and Hollingshead social class score (51.6 vs. 54.8). As expected, the two groups differed greatly in birth weight (1132 vs. 3297 g; *p* < 0.0005) and gestational age at birth (29.1 vs. 38.3 weeks; *p* < 0.0005).

**Table 1:** Sample characteristics (clinical and socio-demographic variables)

	Mean	Mean	*p value*
	(SD)	(SD)	
	95 % CI	95 % CI	
Total enrolled infants	VLBW	FTI	*χ* ^2^ = 0.0275; n.s.
Gender	*n* = 150	*n* = 145	
Female (%)	52.0	51.0	
Male (%)	48.0	48.9	
Birth weight (grams)	1,132	3,297	t = 59.86; *p* < 0.0005
	(232.6)	(374.4)	
	1,094−1,169	3,236−3,357	
Gestational age at birth (weeks)	29.1	38.3	t = 49.13; *p* < 0.0005
	(2.2)	(1.1)	
	28.7−29.5	38.1−38.5	
NICU length of stay (days)	59.7	0	t = 31.55; *p* < 0.0005
	(21.6)	0	
	56.2−63.2		
Mother:			
Socio-economic status (%)	*n* = 148	*n* = 143	
Education (years)	12.5	12.7	t = −0.14; n.s.
	(3.8)	(3.7)	
	11.9−13.1	12.1−13.3	
Father:			
Socio-economic status (%)	*n* = 146	*n* = 142	
Education (years)	12.5	11.5	t = −0.83; n.s.
	(4.0)	(3.4)	
	11.9−13.1	10.9−12.1	
Parents’ socio-economic status score	*n* = 123	*n* = 116	
	51.6	54.8	t = −0.48; n.s.
	(19.9)	(20.5)	
	48.1−55.1	51.1−58.5	

### Birth hospitalisation period

For the VLBWIs, the average length of the initial hospitalisation was 59.7 days (SD 21.6 days), while for the FTIs, it was 3.0 days (SD 0.4 days). The mean cost of the hospitalisation for the VLBWIs was €20,502 (SD €8409). The average cost of hospital staff accounted for 73.9 % of the total cost. Drugs and diagnostic procedures accounted for only 4.1 % and 2.1 %, respectively, of the total cost. The mean total societal cost per infant during the hospitalisation period was €32,460, with the hospital cost accounting for 63.2 % of the total. Travel expenses and productivity losses accounted for 7.2 % and 29.6 % of the total societal cost, respectively. In the control group, the mean total societal cost per infant was €2640; for these patients, the hospitalisation, travel and productivity losses accounted for 34.3 %, 1.6 % and 63.1 % of the cost, respectively (Table 2). The hospital cost and travelling cost were 23 and 57 times higher, respectively, for VLBWIs compared with the control group. The productivity losses due to the delivery hospitalisation (€9606) were much larger for the VLBWIs than for the control group (€1692) due to the much longer hospital stay of the infant.

**Table 2:** Costs from birth to discharge (€)

*Costs*	Infants (n)	User %	Mean cost per infant (SD)	*p* value
				(costs)
Initial hospitalisation (birth)	295	100		
Very low birth weight infants (LOS 59.7; SD 21.6)	150	100	20,502 (8409)	t = 28.66
				*p* < 0.0005
Full-term infants (LOS 3.0; SD 0.4)	145	100	907 (324)	
Travelling	278	100		
Very low birth weight infants	140	100	2352 (3143)	t = 8.8
				*p* < 0.0005
Full-term infants	138	100	41 (63)	
Productivity losses	295	100		
Very low birth weight infants	150	100	9606 (4396)	t = 21.88
				*p* < 0.0005
Full-term infants	145	100	1692 (1048)	
Total societal cost of initial hospitalisation (from birth to discharge)	295	100		
Very low birth weight infants	150	100	32,460 (9136)	t = 35.56
				*p* < 0.0005
Full-term infants	145	100	2640 (826)	

### From discharge to six months (T_0_-T_6_)

A total of 48 VLBWIs (32 %) and 15 FTIs (10.3 %) were re-hospitalised during the first six months after discharge (Table 3). The mean number of re-hospitalisations per VLBWI during the period was 0.4, and the mean length of stay was 4.0 days. Overall, the mean cost of VLBWIs to the Italian SSN in the first six months after discharge was €1433, with re-hospitalisation and drugs accounting for 56.0 % and 19.6 %, respectively, of the total cost. The cost of care borne by the Italian SSN for FTIs was €565, with re-hospitalisation accounting for 71.1 % of the total cost. All costs borne by the Italian SSN were significantly higher in the VLBWI group compared with the control group. There was no difference between the two groups concerning paediatrician visits, other specialist visits, laboratory tests, imaging, and rehabilitation services paid by the families. However, the pharmaceutical care paid by families was significantly higher in the VLBWIs (€269 vs. €69, *p* < 0.0005). The paid and informal care and the productivity losses were not significantly different between the two groups. However, the families of VLBWIs had higher costs for travelling (€96 vs. €56; *p* = 0.0001). The overall mean societal cost for the first six months after discharge was €13,382 for the VLBWIs and €12,368 for the FTIs, with the difference mainly due to hospital and drug costs borne by the Italian SSN.

**Table 3:** Total cost (€) during the first six months after discharge for the families of VLBWIs and FTIs (T_0_-T_6_)

*Costs funded by the Public Health System*	VLBWIs	VLBWIs	VLBWIs	FTIs	FTIs	FTIs	*p* value
	n =150	n = 150	n = 150	n = 145	n = 145	n = 145	(costs)
	Resource users %	Resource consumption Mean	Mean cost (SD)	Resource users %	Resource consumption Mean	Mean cost (SD)	
Other hospitalisations	32.0	0.4	803 (1673)	10.3	0.1	401 (1572)	t = 2.21
							*p* = 0.03
General practitioner consultation (Paediatric)	98.6	4.9	71 (52)	98.6	4.5	64 (35)	t = 1.24
							n.s
Specialist Visits (Follow-up as an outpatient at the birth hospital)	99.3	4.3	90 (59)	57.2	0.9	19 (22)	t = 13.61
							*p* < 0.0005
Other specialist visits	72.6	2.5	53 (57)	18.6	0.2	4 (10)	t = 10.06
							*p* < 0.0005
Drugs	76.0		281 (211)	63.4		70 (79)	t = 4.11
							*p* = 0.0001
Laboratory tests	55.3	3.2	14 (23)	12.4	0.4	2 (9)	t = 6.21
							*p* < 0.0005
Imaging tests	73.3	2.2	106 (114)	10.3	0.1	5 (20)	t = 10.39
							*p* < 0.0005
Rehabilitation therapy	20.0	0.9	10 (23)	0.7	0.0	0.0 (0.0)	t = 4.09
							*p* < 0.0005
Psychological support	7.6	0.3	5 (24)	0.0	0.0	0.0	t = 2.53
							*p* = 0.01
Total cost funded by the Public Health System			1433 (1256)			565 (130)	t = 3.71
							*p* = 0.0002
*Costs funded by Families*							
Drugs	17.0		269 (648)	22.1		69 (42)	t = 4.89
							*p* < 0.0005
Paediatric visits	30.6	1.0	57 (124)	20.7	0.5	54 (41)	t = 0.20
							n.s
Other specialists visits	6.0	0.1	5 (27)	4.8	0.1	4 (27)	t = 0.22
							n.s
Laboratory and imaging tests	2.6	0.0	1 (12)	3.4	0.0	2 (10)	t = −0.47
							n.s
Rehabilitation therapy	0.6	0.0	0.0	0.0	0.0	0.00	-
Paid informal care	33.3		163 (873)	34.7		221 (1192)	t = −0.48
							n.s
Unpaid informal care	28.0		1110 (2431)	28.0		1016 (2196)	t = 0.35
							n.s
Travelling	89.3	65.6 (travels)	96 (111)	87.6	2.8 (travels)	56 (43)	t = 3.97
							p = 0.0001
Productivity losses	66.9		10,246 (6073)	63.8		10,380 (5960)	t = −0.19
							n.s
Total cost funded by families and productivity losses			11,948 (3563)			11,802 (3623)	t = 0.26
							n.s
Total societal cost (T_0_-T_6_)			13,382 (2461)			12,368 (2506)	t = 1.15
							n.s

### From six months to 18 months (T6-T18)

During the one-year period from six to 18 months after the initial hospitalisation, the cost borne by the Italian SSN for VLBWIs remained remarkably higher than the cost of the controls (€820 vs. €391; *p* = 0.0002) (Table 4). With the exception of general paediatric services, all categories of medical costs were higher for the VLBWIs compared to the FTIs. However, the cost of this period was substantially lower than that in the six months following the first hospitalisation. On average, the VLBWIs had a daily SSN medical cost of €12.4 in the first six months after discharge and €3.2 in the following 12 months.

**Table 4:** Total cost (€) from six months to 18 months after discharge for the families of VLBWIs and FTIs (T_6_-T_18_)

*Costs funded by the Public Health System*	VLBWIs	VLBWIs	VLBWIs	FTIs	FTIs	FTIs	*p* value
	*n* = 150	*n* = 150	*n* = 150	*n* = 145	*n* = 145	*n* = 145	(costs)
	Resource users %	Resource consumption mean	Mean cost (SD)	Resource users %	Resource consumption mean	Mean cost (SD)	
Other hospitalisations	23.3	0.4	466 (1147)	6.9	0.1	265 (1,188)	t = 3.06
							*p* < 0.0005
General practitioner consultation (Paediatric)	98.0	5.4	78 (70)	97.2	5.7	81 (75)	t = −0.35
							n.s
Specialist visits (Follow-up as an outpatient at the birth hospital)	96.6	3.5	74 (49)	53.1	0.9	20 (23)	t = 12.06
							*p* < 0.0005
Other specialist visits	54.0	1.6	33 (50)	15.8	0.2	4 (13)	t = 6.66
							*p* < 0.0005
Drugs	83.3		104 (84)	86.2		10 (54.0)	t = 5.66
							*p* < 0.0005
Laboratory tests	29.3	2.3	10 (22)	19.3	0.6	3 (10)	t = 3.23
							*p* < 0.001
Diagnostic tests	28.0	0.6	33 (77)	8.9	0.1	4 (15)	t = 4.44
							*p* < 0.0005
Rehabilitation therapy	12.6	1.2	13 (52)	1.4	0.3	4 (39)	t = 1.76
							n.s
Psychological support	5.3	0.5	9 (89)	0.0	0.0	0.0	t = 1.25
							n.s
Total cost funded by the public health System			820 (145)			391 (87)	t = 6.20
							*p* < 0.0005
*Costs funded by families*							
Drugs	13.5		109 (956)	15.3		11 (23)	t = 1.34
							n.s
Paediatric visits	30.6	1.1	131 (268)	20.7	0.6	68 (170)	t = 2.43
							*p* = 2.43
Other specialists visits	6.0	0.1	4 (20)	6.2	0.1	9 (44)	t = −1.21
							n.s
Laboratory and diagnostic tests	2.0	0.1	1 (5)	1.4	0.0	0 (0)	t = 0.16
							n.s
Rehabilitation therapy	0.6	0.1	0.0 (0)	0 (0)	0 (0)	0 (0)	-
Paid informal care	43.4		1183 (355)	44.2		564 (257)	t = 1.71
							n.s
Unpaid informal care	40.0		3617 (612)	37.0		3770 (615)	t = 0.35
							n.s
Travelling	70.0	3.0	69 (85)	51.0	1.2	16 (23)	t = 7.23;
							*p* < 0.000
Productivity losses	20.2		6323 (9848)	11.1		4373 (1135)	t = −0.19
							n.s
Total cost funded by families and productivity losses			11,437 (2583)			8811 (2007)	t = 1.85
							(*p* = 0.06)
Total societal cost (T_6_-T_18_)			12,257 (1896)			9202 (1476)	t = 2.27
							*p* < 0.02

In this period, only paid and unpaid care and productivity losses were relevant costs borne by the families. Although the differences were not significant, productivity losses tended to be higher for the families of the VLBWIs compared to the families of the FTIs (€6323 vs. €4373, n.s.), while the societal cost due to informal care was similar between the groups (€4790 vs. €4334, n.s). The total societal costs over this 12-month period were €12,257 for the VLBWIs and €9202 for the control group (n.s.), with the difference mainly due to productivity losses and paid care.

### Overall costs

**Fig. 1:**
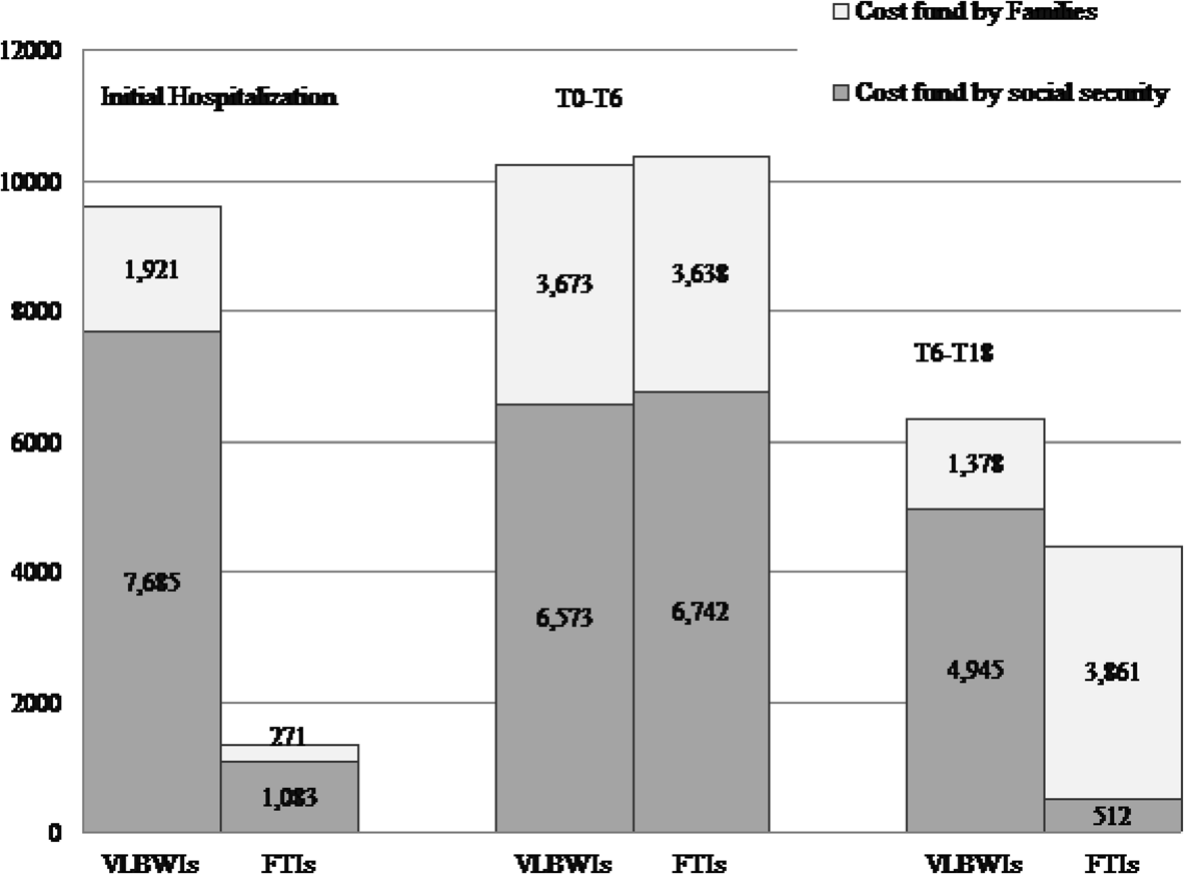
Productivity losses (€) from birth up to 18 months after the infant’s discharge

The mean total cost per VLBWI from birth to the 18th month after discharge was €58,098, while the corresponding cost per FTI was €24,209. The medical costs declined over time for both groups. During the observation period, 62.6 % of the medical costs occurred in the most costly patients’ quartile and only 4.6 % occurred in the least expensive quartile (Fig. 1). Among VLBWIs, the correlation between birth hospitalisation cost and birth weight was significant (r^2^ = −0.20; *p* < 0.0005). The following correlations were also significant: birth hospitalisation cost and gestational age (r^2^ = −0.24; *p* < 0.0005), Length of Stay (LOS) and birth weight (r^2^ = −0.61; *p* < 0.0005), and LOS and gestational age (r^2^ = −0.57; *p* < 0.0005).

**Fig. 2:**
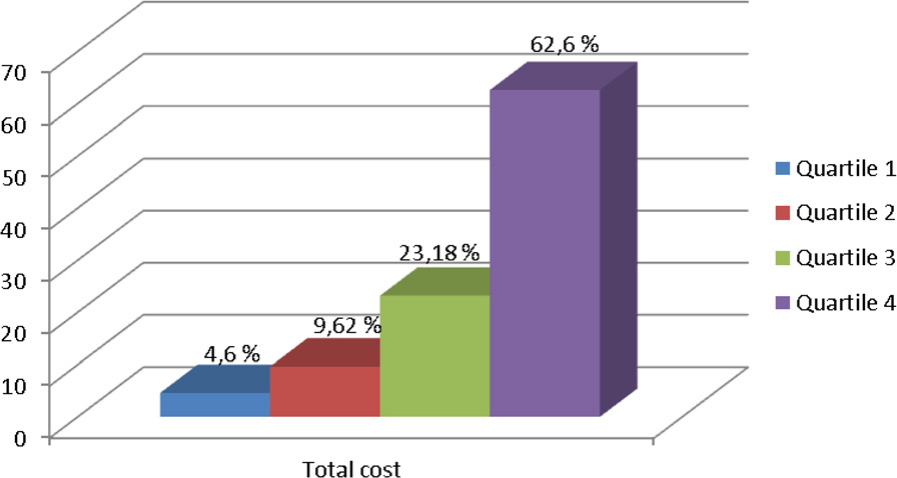
Healthcare cost distribution during the observation period

The non-medical costs had a different pattern. For the VLBWIs, the non-medical costs were higher during the delivery hospitalisation because of the longer stay. The productivity losses tended to be similar between the two groups in the six months after discharge because the maternity leave for the mothers of the VLBWIs was augmented by the period spent in the hospital. In the third period of observation (from six to 18 months after discharge), the prodcpeuctivity losses and paid care were higher for the families with VLBWIs, thus making non-medical costs significantly higher (€11,437 vs. €8881 for families of FTIs; *p* < 0.0005). The productivity losses were mainly borne by social security during the hospitalisation and the first six months after discharge (Fig. 2). Particularly, in the second period of observation (six months after discharge), the proportion of the productivity cost borne by social security was similar (approximately 65 %). However, in the third period (from six months to 18 months after discharge), the families with the FTIs suffered significantly higher direct productivity losses because they did not benefit from extra parental leave due to the condition of the infant. Overall, the total productivity losses for the entire observation period amounted to €19,203 and €8337 for VLBWI and FTI families, respectively.

Of the 106 mothers of VLBWIs whose employment status was available at the end of the observation period, 22 had lost their jobs (20.8 %). The corresponding figure for the 99 mothers of FTIs was 15 (15.2 %). In total, 4.6 % and 0.9 % of mothers of VLBWIs and FTIs, respectively, remained on maternity leave without income.

## Discussion

Our study population included 150 “healthy” VLBWIs born at one of 25 hospitals located in eight different Italian regions and admitted to the internal NICU. In this study, we compared this cohort of infants with a matched cohort of FTIs to report additional medical and societal costs attributable to very low birth weight and early gestational age. As best we know, this is the first study investigating the cost of premature babies in Italy that was based on primary data and is one of the very few studies on the cost of premature babies performed in non-Anglo-Saxon countries. Most studies on the cost of preterm babies have focused on the neonatal intensive care costs and hospital costs during the birth hospitalisation [32]. In contrast, our study included all of the costs related to the infant’s care during the first 18 months after birth discharge and documented paid and informal care and productivity losses. We collected data related to each hospitalisation, enabling us to attribute a monetary value to all the medical resources used to estimate the actual cost of each VLBWI’s hospital stay. The cost borne by the Italian Ssn was primarily calculated on the basis of information from the medical charts by physicians and other information collected from the administrative offices in each hospital. The cost borne by families were estimated based on questionnaires administered to the parents at three different points in time.

For the birth hospitalisation of VLBWIs, we could not use DRGs because they do not reflect actual costs, which our study confirms [33]. Using actual national tariffs, our estimates for the mean cost for the birth admission would have been €4833, with a range between €1987 and €17,162; instead, according to our estimates, the mean cost was €20,502, with a range between €7852 and €47,162.

Previous studies have shown that the cost of the initial hospitalisation in very preterm infants is high [22, 34, 35] and that the cost increases with decreasing gestational age and birth weight and with increasing length of hospital stay. Our results fully confirm these findings. Our data suggest that among VLBWIs, both low birth weight and gestational age are correlated with the length of stay of the index hospitalisation (r^2^ = 0.61 and r^2^ = 0.57, respectively; *p* < 0.0005) and that when birth weight and gestational age are used in the same model, they explain 73 % of variability in the length of stay. This finding suggests that these variables may be used to design tariff systems that better correlate costs than the present, limited numbers of DRGs available for premature babies.

In comparison to previous studies from the US, the UK, Sweden, and Finland, our costs (in 2008 currency) for the initial hospital stay were lower; costs for VLBWIs in other studies have been between €38,660 (2008 currency) and €116,180 (2006 currency). However, our study focused on VLBWIs without prematurity-related morbidities, which can cause significant additional costs. Prematurity-related complications, such as infections and pulmonary problems, may prolong the initial hospitalisation, resulting in additional costs [36]. Our results support the conclusions of Koveranska et al. [37], who made a distinction between preterm infants with and without prematurity-related morbidities. Infants who were born very preterm without prematurity-related morbidities, as in our study population, did not cause significant additional costs for the healthcare service after the initial hospitalisation compared with infants who were born healthy at term. In contrast, individuals with prematurity-related morbidities not only consume more health care resources but also have higher long-term costs [37].

In previous studies, the mean LOS for very preterm infants varied from 41 to 64 days [20–22, 24, 38–40]. Most of these studies report a median LOS shorter than the mean LOS, indicating the presence of a small number of outliers who use a large amount of resources [20–22]. In agreement with such evidence, our study found a mean LOS of 59.7 days and a median LOS of 47 days.

The total cost of premature individuals decreases over time, and the initial hospitalisation composes the largest cost burden [23, 24, 26, 37, 40]. In agreement with these studies, in our study, the cost of the birth hospitalisation for VLBWIs accounted for 89 % of the total healthcare cost and 60 % of the total societal cost over the observation period. After the initial hospitalisation, the healthcare cost remained higher for VLBWIs compared to FTIs, primarily due to re-hospitalisations and pharmaceutical therapies. These costs declined over time and tended to converge in the last observation period (from six to 18 months after discharge). It is important to highlight that the absolute values of these costs are modest (€820 and €391 for VLBWIs and FTIs, respectively) and thus cannot radically modify the cost-effectiveness profile of interventions during the first hospitalisation and following periods. This finding confirms the crucial role of neonatal care received during birth hospitalisation and suggests that attempts to improve quality and cost-effectiveness of interventions for these infants should be focused during this phase [32].

In our study, the paid care and informal care costs were similar between the two groups in the first six months after discharge, primarily because mothers (and less fathers) benefited from parental leaves and thus could directly take care of the infant. However, in the following year, the additional cost of paid care for the families with VLBWIs was more than two-fold higher than the cost for the parents of FTIs, which created an additional economic burden that was even higher than the additional cost for healthcare. In contrast, in this period, the unpaid infant care was the same between the preterm and the control group. This divergent pattern suggests that paid care is additional and not substitutive to informal care.

The families of VLBWIs suffered from higher productivity losses because they had fewer working days, and the mothers were also more likely to lose their jobs. These findings are consistent with studies from other European countries, which were recently summarised in a review of the existing literature by Hodek et al. [41], who illustrated the significant burden of prematurity on parents.

To appreciate the societal implications of paid and informal care and productivity losses, it is important to separate private costs from social security costs. In the first periods (during the initial hospitalisation and the first months after the delivery), all parents receive social security support in the form of parental leave. Afterwards, the mothers of VLBWIs seem to benefit from additional support provided by social security in the form of prolonged parental leaves (although with income reduction). For the families of FTIs, such benefits are much more limited; the mothers are more frequently back at work, but they also often have to reduce their working hours and miss working days to care for their infants. This fact may explain why in this period mothers of these infants suffer higher economic losses than the mothers of VLBWIs.

This evidence must be interpreted in the specific context of the Italian society, where female participation to the labour market is low compared with the rest of Europe. Infant care is provided by the parents (mainly mothers) and unpaid caregivers, while the cost borne for paid care is limited. Interestingly, there is no difference between the two groups in the fraction of infants receiving unpaid care and the average time spent per infant, thus suggesting that in Italy, informal, unpaid care provided by relatives is crucial for preterm infants.

The major strength of our study is the use of a national cohort of VLBWIs as a study population. The use of this population avoids the selection bias that characterises studies that are based in smaller geographic regions or a single hospital. We used data on true hospital admissions. Moreover, we took into account the family perspective when calculating the overall cost of preterm births, and we quantified the main categories that should be evaluated to adequately measure the burden of preterm birth on families. However, many of the adverse consequences of preterm birth, such as cognitive and behavioural problems, are likely to be diagnosed later; consequently, the effect of these conditions on later costs should be studied at an older age. Thus, a limitation of this study is that the data on the resources used were limited to 18 months of age.

## Conclusions

The present study showed that for VLBWIs born in Italy, the initial hospitalisation accounted for the clear majority of the healthcare cost during the first 18 months of life. However, a minority of children born very preterm had a long initial LOS and more re-admissions and outpatient visits during the follow-up period, resulting in higher healthcare costs for this group. A reduced hospital length of stay, a higher gestational age and an increasing birth weight are likely to result in cost savings and thus improve the cost-effectiveness of care. The further prevention of morbidities during the prenatal period and increased quality of care provided by NICUs to adequately manage such conditions, would therefore significantly reduce the cost of prematurity. Our findings highlight that the existing DRGs and relative tariffs in use in Italy to fund NICUs inadequately reflect the NICU’s actual costs.

In addition, our study suggests that when estimating the cost of prematurity after the initial hospitalisation, one should not only calculate healthcare costs but also the burden on the families and caregivers of these infants because these costs greatly exceed the healthcare cost for VLBWIs after the initial discharge. The burden of very low birth weight goes far beyond the cost that the National Health Service covers because the birth of a preterm infant may also have an economic impact over time on other parties. Decision makers and healthcare providers should be aware of the total cost supported by society when considering different sources of expenditure to design policies targeted to a reduction and a fair distribution of the total societal burden and not on the healthcare burden alone.

## References

[1] Ministero della Salute. Dipartimento della Qualità, Direzione Generale del Sistema Informativo, Ufficio Direzione Statistica. Certificato di assistenza al parto (CeDAP) - Analisi dell’evento nascita - Anno 2008, www.salute.gov.it; marzo 2011.

[2] Roberts D, Dalziel SR (2006). Antenatal corticosteroids for accelerating fetal lung maturation for women at risk of preterm birth. Cochrane Database Syst Rev.

[3] Soll RF (1998). Surfactant treatment of the very preterm infant. Biol Neonate.

[4] Gultom E, Doyle LW, Davis P, Dharmalingam A, Bowman E (1997). Changes over time in attitudes to treatment and survival rates for extremely preterm infants (23–27 weeks’ gestational age). Aust N Z J Obstet Gynaecol.

[5] Doyle LW, Rogerson S, Chuang SL, James M, Bowman ED, Davis PG (1999). Why do preterm infants die in the 1990s?. Med J Aust.

[6] Doyle LW (2004). Victorian Infant Collaborative Study Group. Evaluation of neonatal intensive care for extremely low birth weight infants in Victoria over two decades: I. Effectiveness. Pediatrics.

[7] Fanaroff AA, Stoll BJ, Wright LL, Carlo WA, Ehrenkranz RA, Stark AR (2004). Trends in neonatal morbidity and mortality for very low birthweight infants. Am J Obstet Gynecol.

[8] Slattery MM, Morrison JJ (2002). Preterm delivery. Lancet.

[9] Gäddlin PO, Finnström O, Wang C, Leijon I (2008). A fifteen-year follow-up of neurological conditions in VLBW children without overt disability: relation to gender, neonatal risk factors, and end stage MRI findings. Early Hum Dev.

[10] Bracewell M, Marlow N (2002). Patterns of motor disability in very preterm children. Ment Retard Dev Disabil Res Rev.

[11] Wolke D, Meyer R (1999). Cognitive status, language attainment, and prereading skills of 6-year-old very preterm children and their peers: the Bavarian Longitudinal Study. Dev Med Child Neurol.

[12] Botting N, Powls A, Cooke RW, Marlow N (1998). Cognitive and educational outcome of very-low-birthweight children in early adolescence. Dev Med Child Neurol.

[13] Hall A, McLeod A, Coulsell C, Thomson L, Mutch L (1995). School attainment, cognitive ability and motor function in a total Scottish very-low-birthweight population at eight years: a controlled study. Dev Med Child Neurol.

[14] Horwood LJ, Mogridge N, Darlow BA (1998). Cognitive, educational, and behavioural outcomes at 7 to 8 years in a national very low birthweight cohort. Arch Dis Child Fetal Neonatal Ed.

[15] Botting N, Powls A, Cooke RW, Marlow N (1997). Attention deficit hyperactivity disorders and other psychiatric outcomes in very low birthweight children at 12 years. J Child Psychol Psychiatry.

[16] Wolke D (1998). Psychological development of prematurely born children. Arch Dis Child.

[17] Pharoah PO, Stevenson CJ, Cooke RW, Stevenson RC (1994). Prevalence of behavior disorders in low birthweight infants. Arch Dis Child.

[18] Hack M, Youngstrom EA, Cartar L, Schluchter M, Taylor HG, Flannery D (2004). Behavioral outcomes and evidence of psychopathology among very low birth weight infants at age 20 years. Pediatrics.

[19] Petrou S, Eddama O, Mangham L (2010). A structured review of the recent literature on the economic consequences of preterm birth. Arch Dis Child Fetal Neonatal Ed.

[20] Phibbs CS, Schmitt SK (2006). Estimates of the cost and length of stay changes that can be attributed to one-week increases in gestational age for premature infants. Early Hum Dev.

[21] Ringborg A, Berg J, Norman M, Westgren M, Jonsson B (2006). (2006) Preterm birth in Sweden: What are the average lengths of hospital stay and the associated inpatient costs?. Acta Paediatr.

[22] Gilbert WM, Nesbitt TS, Danielsen B (2003). The cost of prematurity: quantification by gestational age and birth weight. Obstetrics Gynecol.

[23] Gray D, Woodward LJ, Spencer C, Inder TE, Austin NC (2006). Health service utilisation of a regional cohort of very preterm infants over the first 2 years of life. J Paediatr Child Health.

[24] Leijon I, Finnström O, Sydsjö G, Wadsby M (2003). Use of healthcare resources, family function, and socioeconomic support during the first four years after preterm birth. Arch Dis Childhood Fetal Neonatal Ed.

[25] Elder DE, Hagan R, Evans SF, Benninger HR (1999). and French N.P. Hospital admissions in the first year of life in very preterm infants. J Paediatr Child Health.

[26] Petrou S, Mehta Z, Hockley C, Cook-Mozaffari P, Henderson J, Goldacre M (2003). The impact of preterm birth on hospital inpatient admissions and costs during the first 5 years of life. Pediatrics.

[27] Montirosso R, Del Prete A, Bellù R, Tronick E, Borgatti R. Level of NICU Quality of Developmental Care and Neurobehavioral Performance in Very Preterm Infants. Pediatrics 2012; 129: 1–9 (in press). 10.1542/peds.2011-0813 PMC4074610 22492762

[28] Hollingshead AB (1975). Four factor index of social status. Unpublished manuscript.

[29] ACI. Automobile Club d’Italia. Calcolo dei costi chilometrici, www.aci.it; 2011.

[30] Ferrovie dello Stato, www.trenitalia.com; 2011.

[31] Banca d’Italia. Eurosistema. Supplemento al Bollettino Statistico. Indagini campionarie. I bilanci delle famiglie italiane nell’anno 2008. Anno XX. N° 8. Febbraio 2010, www.bancaditalia.it.

[32] Zupancic JAF. A Systematic Review of Costs Associated with Preterm Birth. In: Behrman RE, Stith Butler A, editors. Preterm birth: Causes, consequences, and prevention, www.nap.edu/catalog/11622.html; 2007.

[33] Fattore G, Torbica A (2006). Inpatient reimbursement system in Italy: How do tariffs relate to costs?. Health Care Manage Sci.

[34] Schmitt SK, Sneed L, Phibbs CS (2006). Costs of newborn care in California: a population based study. Pediatrics.

[35] Rogowski J (1999). Measuring the cost of neonatal and perinatal care. Pediatrics.

[36] Russell RB, Green NS, Steiner CA, Meikle S, Howse JL, Poschman K (2007). Cost of hospitalization for preterm and low birth weight infants in the United States. Pediatrics.

[37] Korvenranta E, Lehtonen L, Rautava L, Kākkinen U, Andersson S, Gissler M (2010). Impact of Very Preterm Birth on Health Care Costs at Five Years of Age. Pediatrics.

[38] Korvenranta E, Linna M, Rautava L, Andersson S, Gissler M, Hallman M (2010). Hospital costs and quality of life during 4 years after very preterm birth. Arch Pediatr Adolesc Med.

[39] St John EB, Nelson KG, Cliver SP, Bishnoi RR, Goldenberg RL (2000). Cost of neonatal care according to gestational age at birth and survival status. AmJ Obstet Gynecol.

[40] Rogowski J (1998). Cost-effectiveness of care for very low birth weight infants. Pediatrics.

[41] Hodek JM, von der Schulenburg JM, Mittendorf T (2011). Measuring economic consequences of preterm birth - Methodological recommendations for the evaluation of personal burden on children and their caregivers. Health Econ Rev.

